# An Unusual Presentation of Acute Pancreatitis With Atrial Fibrillation: A Case Report

**DOI:** 10.7759/cureus.96171

**Published:** 2025-11-05

**Authors:** Sakshin Belgavi, R Rajesh, Ajay Pawar

**Affiliations:** 1 Emergency Medicine, Jawaharlal Nehru Medical College, Belagavi, IND

**Keywords:** acute pancreatitis, acute pancreatitis complications, atrial fibrillation (af), clinical case report, sensorimotor polyneuropathy

## Abstract

Acute pancreatitis (AP) is an inflammatory disorder of the pancreas that can lead to severe systemic complications, including multi-organ failure and systemic inflammatory response syndrome (SIRS). While gastrointestinal and metabolic manifestations are well-documented, cardiovascular and neurological presentations are rare. This case describes an atypical presentation of AP in a young, otherwise healthy male, who initially experienced bilateral ascending lower limb pain and weakness. These symptoms progressed to lower abdominal pain, nausea, vomiting, and breathlessness. An electrocardiogram upon presentation revealed atrial fibrillation. This constellation of signs and symptoms presented a diagnostic challenge; however, subsequent blood tests and imaging confirmed the diagnosis of AP. Despite aggressive management in the intensive care unit, the condition rapidly progressed to multi-organ failure, resulting in death. This case underscores the importance of early recognition of atypical presentations of AP. Physicians, especially those in emergency departments, should maintain a high index of suspicion to enable early intervention and prevent fatal outcomes.

## Introduction

Acute pancreatitis (AP) is an inflammatory condition of the pancreas, varying from mild self-limiting disease to severe necrotizing pancreatitis associated with high morbidity and mortality. The most common causes include gallstones and alcohol use, although idiopathic cases also occur. The diagnosis of AP requires two of the following three criteria: abdominal pain consistent with the disease, serum amylase and/or lipase levels greater than three times the upper limit of normal, and/or characteristic findings from abdominal imaging [1]. Early diagnosis and management of AP are essential to prevent complications [2].

Typical presentations of AP most frequently include epigastric or left upper quadrant abdominal pain associated with nausea and vomiting [1]. Atypical symptoms without abdominal pain may include fever, syncope, and/or dyspnea, which have been less frequently reported [3]. Cardiovascular complications, such as atrial fibrillation (AF), are rare and are often associated with systemic inflammation. Abnormalities of cardiac rhythm, contractility, and vasomotor tone of peripheral vessels are common cardiovascular manifestations. The pathogenetic factors of cardiac manifestations include hypovolemia and metabolic disturbances (eg, hyperkalemia, hypomagnesemia, and hypophosphatemia) [4]. Neurological complications in AP are even less frequently reported and may result from metabolic derangements, electrolyte disturbances, or neuroinflammatory processes [5-7].

We present a case of acute severe pancreatitis in a young male without identifiable risk factors, who initially developed bilateral lower limb weakness and pain, as well as AF, before progressing to multi-organ failure and death within 24 hours of admission.

## Case presentation

A 22-year-old male presented to our Emergency Department (ED), in a tertiary care center, with complaints of bilateral lower limb pain and weakness, lower abdominal pain, vomiting, and loose stools for the past two days. He was referred from a local hospital with suspicion of Guillain-Barré (GB) syndrome, where MRI of the brain and spine was performed and reported to be unremarkable.

The patient reported that he was apparently well two days prior when he developed a sudden onset of pain in both legs, which progressed to the lower abdomen. The pain was dull to burning in nature, non-radiating, and associated with three episodes of vomiting and one episode of loose stools, which were semi-solid in consistency and non-blood-tinged. He reported consuming spicy food and cold carbonated drinks but denied any history of alcohol consumption, including prior to the onset of symptoms. He also gave a history of flu-like symptoms two weeks earlier and had no significant previous medical history or comorbidities. No significant family history or drug allergies were noted.

On examination, the patient was conscious, cooperative, and well-oriented to time, place, and person but was visibly distressed due to pain. Vital signs included a pulse rate of 132 beats/min, which was irregular; blood pressure of 130/80 mmHg; SpO2 of 96% on room air; respiratory rate of 56 breaths/min; and a body temperature of 36°C. Abdominal examination revealed diffuse tenderness, a soft, non-distended abdomen with normal bowel sounds. Neurological examination findings included 5/5 power in all four limbs, sluggish deep tendon reflexes, flexor plantar responses, hyporeflexia, and subjective numbness in both lower limbs.

**Figure 1:**
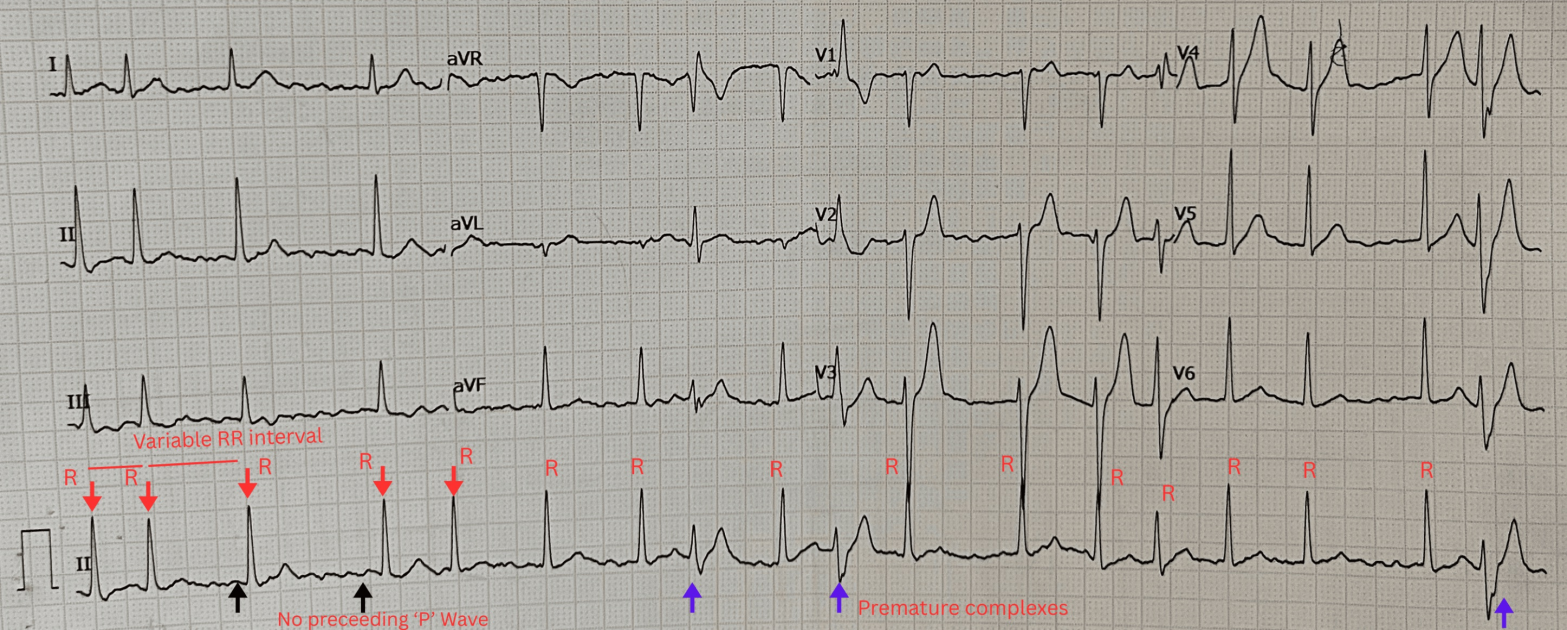
Electrocardiogram showing atrial fibrillation with rapid ventricular response and premature/aberrantly conducted complexes.

The patient was started on intravenous (IV) acetaminophen 1 g, followed by tramadol 100 mg/2 ml diluted in 100 ml of 0.9% NaCl IV infusion. An electrocardiogram showed atrial fibrillation with rapid ventricular response (Figure 1). He was placed on non-invasive ventilation, as arterial blood gas (ABG) revealed primary metabolic acidosis with secondary respiratory alkalosis. Point-of-care ultrasonography (POCUS) of the abdomen indicated an enlarged pancreas, prompting investigations into the possibility of AP. Contrast-enhanced computed tomography (CECT) of the abdomen and pelvis showed acute interstitial edematous pancreatitis with peripancreatic fluid collection. POCUS 2D echocardiography revealed no regional wall motion abnormality and preserved ejection fraction. Blood tests revealed serum amylase levels of 1568 U/L (normal range: 30-110 U/L); serum lipase levels of 4395 U/L (normal range: 13-60 U/L); normal serum triglyceride levels of 30 mg/dL (normal levels <150 mg/dL); normal serum troponin I; and a total leukocyte count of 17,450 per µL (Table 1).

**Table 1:** Laboratory tests on admission and the second day AST: aspartate aminotransferase; ALT: alanine aminotransferase; ALP: alkaline phosphatase; INR: International Normalized Ratio; HDL: high-density lipoprotein; LDL: low-density lipoprotein All the above values are for serum/blood tests. '-' indicates that the laboratory test has not been performed on the specified day.

Parameter	Day 1	Day 2	Reference values
Hemoglobin (g/L)	18.2	-	13.5 – 17.5
White Blood Cells (/µL)	17600	-	4000 – 11000
Platelets (/µL)	413000	-	150000 – 500000
Total bilirubin (mg/dl)	2.44	6.96	0.0 – 1.4
Direct bilirubin (mg/dl)	0.78	4.84	0.0 – 0.3
Indirect bilirubin (mg/dl)	1.66	2.12	0.0 – 1.1
ALT (IU/L)	144	473	1 – 41
AST (IU/L)	234	269	1 – 40
ALP (IU/L)	92	98	40 – 130
Albumin (g/dl)	4.2	4.1	3.5 – 5.2
Amylase (U/L)	1568	-	28 – 100
Lipase (U/L)	4395	-	13 – 60
Creatinine (mg/dl)	1.81	3.93	0.60 – 1.17
Sodium (mmol/L)	133	131	136 – 145
Potassium (mmol/L)	4.17	4.89	3.5 – 5.1
Calcium (mg/dl)	8.2	-	8.6 – 10.2
INR	1.36	-	0.86 – 1.12
Total cholesterol (mg/dl)	49	-	<200
LDL cholesterol (mg/dl)	16	-	<100
HDL cholesterol (mg/dl)	32	-	40 – 60
Triglycerides (mg/dl)	30	-	<150

The patient was admitted to the intensive care unit and started on intravenous fluids (Ringer's lactate at 125 ml/hour), antibiotics for infection control (intravenous meropenem 1 gram every eight hours), and adequate analgesics. The AF spontaneously resolved and reverted to sinus rhythm two hours after admission. Despite intensive care, the patient’s condition continued to deteriorate with worsening renal and liver function parameters. He developed respiratory failure following an episode of hypoxia, for which he was intubated and placed on mechanical ventilation. Subsequently, the patient developed hypotension and was started on vasopressors. He suffered a cardiac arrest, and despite resuscitative efforts, he could not be revived and was declared dead 24 hours after admission. The cause of death was determined to be multi-organ failure secondary to acute pancreatitis.

## Discussion

This case illustrates a rare presentation of AP in a young patient. The sudden onset of ascending weakness and pain from the lower limbs suggested GB syndrome as one of the main diagnoses that had to be considered in the earlier stages. AF on presentation further added to the confusion, as mesenteric ischemia would have been another legitimate differential as symptoms progressed to abdominal pain. Atypical AP, defined as any symptomatology in the absence of abdominal pain, is generally related to pre-existing systemic diseases (e.g., lupus erythematosus), advanced age, and rarely as a post-operative subclinical complication [3]. Diagnosis in these cases occurs accidentally and is mainly based on biochemical abnormalities or characteristic radiologic results performed for other clinical reasons [3]. The metabolic acidosis, elevated pancreatic enzymes, and CECT findings confirmed the diagnosis of AP.

Even though AF has been reported in AP in the past, this finding is rare in a young patient with no pre-existing cardiovascular or other comorbidities [8,9]. The pathogenic factors of cardiovascular manifestations include hypovolemia and metabolic disturbances (e.g., hyperkalemia, hypomagnesemia, and hypophosphatemia) [4]. Although cardiac manifestations are reversible with appropriate management, the presence of AF in patients with AP increases mortality and leads to worse clinical outcomes [4,10]. Neurological complications of AP are even more rare. Reports of pancreatic encephalopathy and acute sensorimotor polyneuropathy as complications of AP are generally not seen at initial presentation but have occurred days to weeks later [5-7]. Neurological involvement may have been secondary to metabolic dysfunction and systemic inflammatory response syndrome [11].

However, this case has several limitations. Being a single-patient report, its findings may not be generalizable to broader populations. Additionally, the pathophysiological link between AP, neurological symptoms, and AF remains unclear due to the absence of cerebrospinal fluid (CSF) analysis, electromyography (EMG), or nerve conduction studies (NCS). The rapid disease progression within 24 hours restricted additional diagnostic and therapeutic interventions, making it difficult to explore alternative management strategies.

Despite these limitations, this case expands the spectrum of AP presentations and highlights the importance of recognizing atypical symptoms early. Future research is needed to explore the underlying mechanisms of neurological and cardiac involvement in pancreatitis, as well as to develop improved management strategies for such severe presentations.

## Conclusions

This case brings attention to the rare and multifaceted presentations of AP, showcasing the importance of swift action and expansive thinking in tackling complex diagnoses. The rapid decline of this young patient, beginning with unusual symptoms like bilateral limb weakness and culminating in multi-organ failure, serves as a stark reminder of the unpredictable nature of AP. It also underscores the need to identify atypical symptoms early, even in the absence of classic risk factors, to improve patient outcomes.

This case is a compelling argument for continued research into the links between AP and rare complications such as atrial fibrillation and neurological manifestations. For medical professionals, it emphasizes the importance of vigilance and adaptability, as unconventional details can often hold the key to a patient’s survival.
